# Effects of Light Intensity on Physiological Characteristics and Expression of Genes in Coumarin Biosynthetic Pathway of *Angelica dahurica*

**DOI:** 10.3390/ijms232415912

**Published:** 2022-12-14

**Authors:** Yongjie Huang, Yongnan Zhai, Ya Huang, Yufeng Huang, Kun Liu, Jie Zhang, Jihai Zhou

**Affiliations:** 1 School of Ecology and Environment, Anhui Normal University, Wuhu 241000, China; 2 Collaborative Innovation Center of Recovery and Reconstruction of Degraded Ecosystem in Wanjiang Basin Co-Founded by Anhui Province and Ministry of Education, Anhui Normal University, Wuhu 241000, China; 3 College of Life Sciences, Anhui Normal University, Wuhu 241000, China

**Keywords:** light intensity, *Angelica dahurica*, physiological characteristics, coumarin biosynthesis, gene expression

## Abstract

Plants are affected by changes in light and adaptation mechanisms can affect secondary metabolite synthesis. In this study, the physiological response and regulation of the coumarin biosynthetic pathway of *Angelica dahurica* to different light intensities (natural light (CK), shade rate 50% (L1), shade rate 70% (L2), and shade rate 90% (L3)) were examined. The chlorophyll content, level of the enzymes of the antioxidant system, extent of lipid peroxidation, and concentrations of the osmoregulatory solute levels were determined in potted plants. Root transcriptome under different light intensities was sequenced using high-throughput technology, and differentially expressed genes (DEGs) related to coumarin biosynthesis were analyzed by quantitative real-time PCR (qRT-PCR). With increasing shade, Chl a, Chl b, Chl a + b, and Chl a/b content increased, while the Chl a/b ratio decreased. The antioxidant enzyme system activity and extent of membrane lipid peroxidation increased. The soluble protein (SP) and proline (Pro) content decreased with the reduction in the light intensity, and soluble sugar (SS) content was found to be highest at 50% shade. The RNA-seq analysis showed that 9388 genes were differentially expressed in the L3 group (7561 were upregulated and 1827 were downregulated). In both the L1 and L2 groups, DEGs were significantly enriched in “Ribosome biosynthesis”; meanwhile, in the L3 group, the DEGs were significantly enriched in “Amino and ribonucleotide sugar metabolism” in KEGG metabolic pathway analysis. Additionally, 4CL (TRINITY_DN40230_c0_g2) and COMT (TRINITY_DN21272_c0_g1) of the phenylpropanoid metabolic pathway were significantly downregulated in the L3 group. In conclusion, *A. dahurica* grew best under 50% shade and the secondary-metabolite coumarin biosynthetic pathway was inhibited by 90% shade, affecting the yield and quality of medicinal compounds.

## 1. Introduction

Light is one of the key environmental factors for plant growth, providing the material and energy basis for plant life activities [1]. Light intensity affects plant morphology and physiology in various ways, including the contents of photosynthetic pigments, as well as the levels of osmotic substances and reactive oxygen species [2,3,4]. Moreover, light intensity affects the expression of genes involved in secondary metabolite synthesis, nutrient uptake, and the allocation of biomass to the roots of plants [5,6,7]. Previous studies by Deng et al. [8] showed that light intensity significantly affected the accumulation of total triterpenoids in the leaves of *Cyclocarya paliurus*. Ghasemzadeh et al. [9] found that light can stimulate the accumulation and distribution of total phenols and total flavonoids in young ginger varieties (*Zingiber officinale Roscoe*). Xu et al. [10] reported that sunlight enhanced flavonoid biosynthesis and promoted the expression of flavonoid-biosynthesis-related genes in the leaves of Ginkgo. Thus, light plays an important role in plant growth and secondary metabolite synthesis, and proper light intensity is important for the growth and quality of medicinal plants.

*Angelica dahurica* (Fisch.ex Hoffm.) Benth. et Hook. f. is an herbal medicinal plant of the Apiaceae family, which is widely grown and distributed in Northeast and North China [11,12]. The root of *A. dahurica* has high medicinal value as one of the commonly used drugs in Chinese medicine clinics and as an important medicinal food plant [13]. The roots are rich in coumarins, volatile oils, flavonoids, trace elements, and alkaloids [14]. It has been shown that coumarins, including simple coumarins, pyranocoumarins, and furanocoumarins, are the most abundant and major secondary metabolites in *A. dahurica* root [15,16] that have antipyretic, analgesic [17], antioxidant [18], anti-inflammatory [19], and antitumor properties [20]. Several enzymes of the phenylpropanoid metabolic pathway are involved in the formation of coumarins [21], including phenylalanine ammonia-lyase (PAL), followed by cinnamic acid-4-hydroxylase (C4H), 4-coumaric acid-CoA ligase (4CL), hydroxycinnamic acid acyltransferase (HCT), p-coumaric acid 3-hydroxylase (C3H), and caffeic acid O-methyltransferase (COMT) [22]. The glossary of abbreviations for professional terms is listed in Table 1.

In recent years, the majority of studies on *A. dahurica* have focused on its chemical composition [14], pharmacology [23], and efficacy [24], and only a few studies have focused on the molecular mechanisms that regulate growth and coumarin biosynthesis by different light intensities. In this study, we examined the physiological characteristics of *A. dahurica* seedlings under different light intensities and used RNA-seq technology to investigate the mechanism of the regulation of genes in the coumarin biosynthesis pathway by light intensity. We aimed to address three questions: (1) What is the optimal light intensity for the growth of *A. dahurica* seedlings? (2) How do different light intensities affect photosynthetic pigments, antioxidant systems, and osmoregulatory substances? (3) How do coumarin biosynthesis genes in *A. dahurica* roots respond to different light intensities? The broader objective of this study was to develop a theoretical foundation for cultivating, managing, and improving various medicinal plants.

## 2. Results

### 2.1. Effect of Light Intensity on Physiological Characteristics of Leaves

#### 2.1.1. Effect of Light Intensity on Photosynthetic Pigments

**Figure 1:**
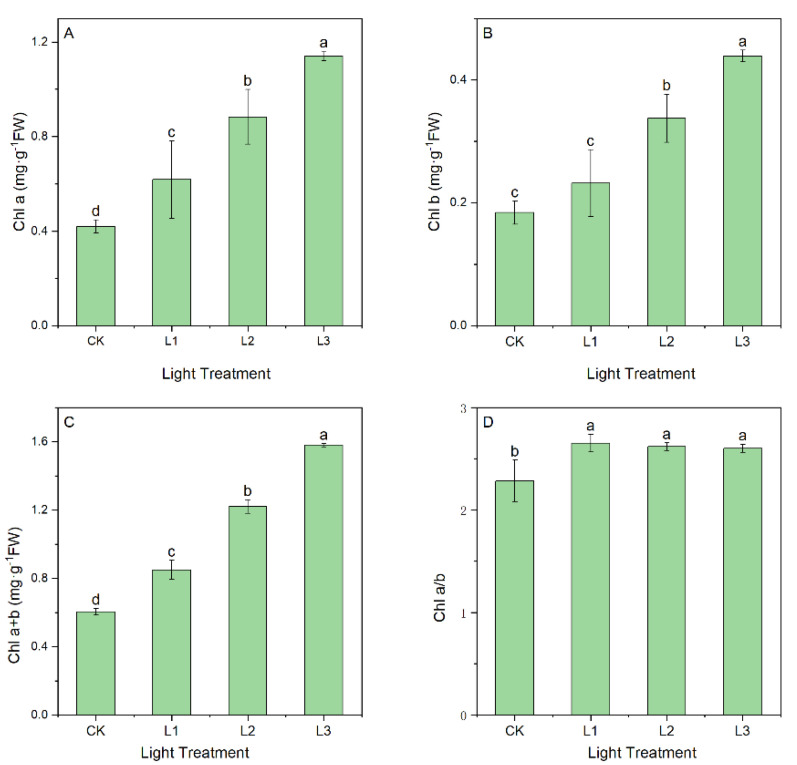
Chl a content (**A**), Chl b content (**B**), Chl a + b content (**C**), and Chl a/b ratio (**D**) in leaves grown under different light intensities. Lowercase letters indicate significant differences under different light intensities (*p <* 0.05).

The contents of photosynthetic pigments were found to be significantly different in leaves under different light intensities (Figure 1). With an increase in shading rate, the contents of Chl a, Chl b, and Chl a + b in the leaves significantly increased (*p* < 0.05), and the Chl a/b ratio showed a trend in which it increased and then decreased compared to the control (*p* < 0.05). The Chl a content of the L1, L2, and L3 plants increased by 47.12%, 110.26%, and 171.26%, respectively, and the Chl b content increased by 26.05%, 83.47%, and 138.50%, respectively (Figure 1A,B). Likewise, the Chl a + b content increased by 40.69%, 102.09%, and 161.53%, respectively (Figure 1C). In addition, the Chl a/b ratios decreased by 15.49%, 13.79%, and 13.28%, respectively (Figure 1D).

#### 2.1.2. Effect of Light Intensity on Antioxidant Enzyme Activity and MDA Content 

**Figure 2:**
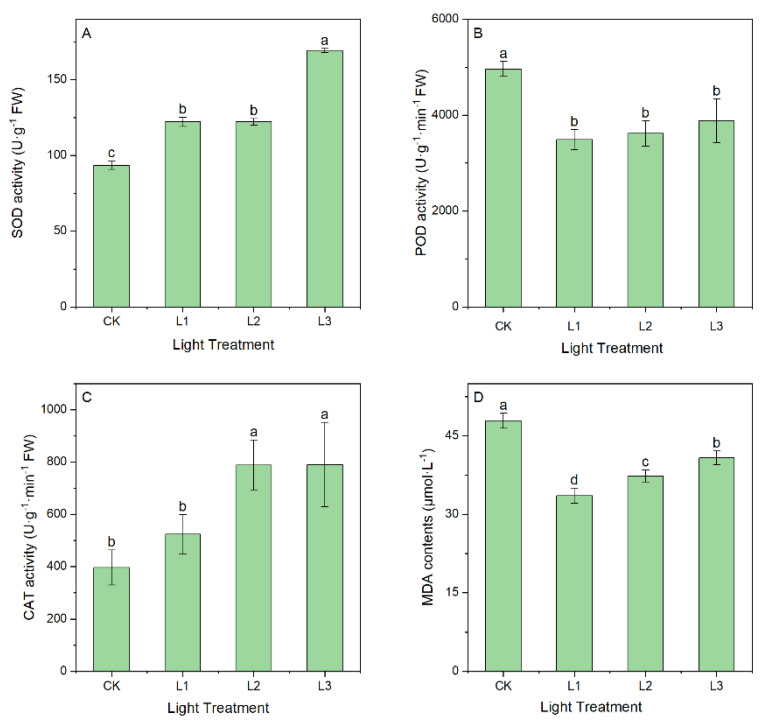
SOD activity (**A**), POD activity (**B**), CAT activity (**C**), and MDA contents (**D**) in leaves grown under different light intensities. Lowercase letters indicate significant differences under different light intensities (*p <* 0.05).

The effect of light intensity on antioxidant enzyme activities in *A. dahurica* seedling leaves is shown in Figure 2. Both SOD and CAT activities were significantly higher in the shade conditions than in the natural light condition (*p <* 0.05). SOD and CAT increased by 81.11% and 99.12% in the L3 condition, respectively (Figure 2A,C). POD activity and MDA content were significantly lower in the shade conditions but an increasing trend with increasing shade was observed (*p <* 0.05) (Figure 2B,D).

#### 2.1.3. Effect of Light Intensity on the Content of Osmoregulatory Substances

**Figure 3:**
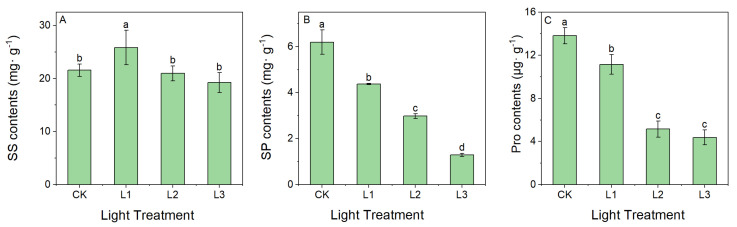
SS contents (**A**), SP contents (**B**), and Pro contents (**C**) in leaves grown under different light intensities. Lowercase letters indicate significant differences under different light intensities (*p <* 0.05).

The effect of different light intensities on osmoregulatory substances in the leaves is shown in Figure 3. Compared with the CK group, the SS content increased in the L1 group, but further decreased in the L2 and L3 groups. The SS content of the leaves of the plants of the L1 group was significantly higher than that of the CK group and the L2 and L3 treatment groups (*p <* 0.05) (Figure 3A). On the other hand, the SP and Pro contents showed an overall trend in the gradual decrease with the degree of shade, and the SP and Pro contents were lowest in the L3 condition (Figure 3B,C). The SP and Pro contents were significantly different between the CK and L3 groups (*p <* 0.05).

### 2.2. RNA-Seq of Root Tissue under Different Light Intensities

#### 2.2.1. De Novo Assembly

We obtained the transcriptome of roots under different shade treatments. A total of 12 samples were sequenced using the Illumina Novaseq 6000 platform and 89.66 Gb of clean read data was obtained. On average, over 6.12 Gb of clean read data was acquired per sample. The percentage of Q30 bases was above 93.57%, and the GC content was not less than 42.31% (Supplementary Table S1). 

Trinity software was used to perform de novo assembly of the clean reads of the sequencing data and to evaluate the optimization (Supplementary Figure S1 and Supplementary Table S2). After assembly, there were 294,362 unigenes, with a total transcript length of 242,418,069 bp. The longest, shortest, and average transcript lengths were 12,050 bp, 201 bp, and 823.54 bp, respectively. The N50 length was 1331 bp, the E90N50 length was 1955 bp, the matching rate was 87.912%, and the GC content was 42.79%. The above figures indicate a high degree of integrity in the assembled fragments.

#### 2.2.2. Functional Annotation of Unigenes

Six databases were used for the annotation of the assembled unigenes (Supplementary Table S3). A total of 95,434 (63.09%), 83,018 (41.02%), 89,172 (44.06%), 70,589 (34.88%), and 100,463 (49.64%) unigenes matched with the Pfam, GO, KEGG, SwissProt, and eggNOG databases, respectively (Supplementary Figures S2 and S3; Supplementary Tables S4–S6). Additionally, the annotated sequences were compared with the 101,839 (50.32%) unigenes in the NR database (Supplementary Figure S4), and the highest sequence similarities were obtained in those of *Daucus carota*, followed by *Quercus suber* (Supplementary Figure S4).

#### 2.2.3. Analysis of Differentially Expressed Genes 

**Figure 4:**
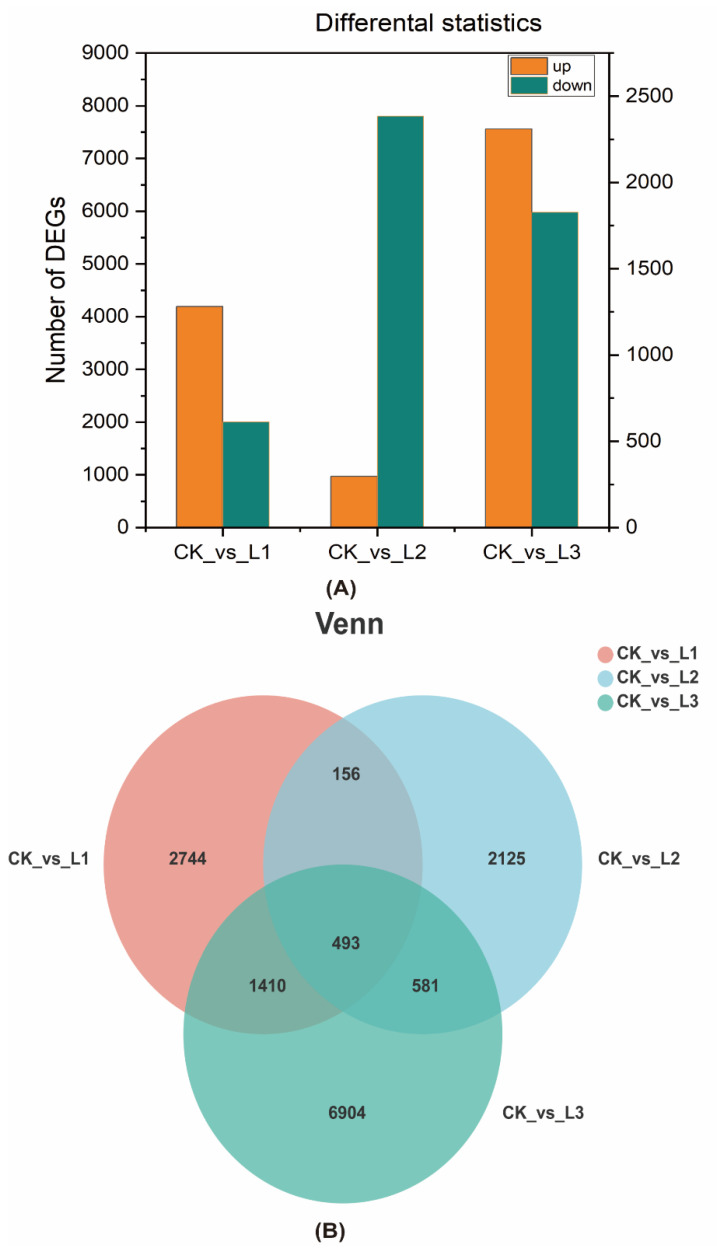
Differentially expressed genes in root tissues of *A. dahurica* under different light intensities. (**A**) Bar graph of different differentially expressed genes. The left y-axis indicates upregulated genes, and the right y-axis indicates downregulated genes. (**B**) Venn diagram analysis of differentially expressed genes.

To reveal the gene expression patterns of roots under different light intensities, the homogenization of sequencing reads was performed. A total of 4803 genes were differentially expressed in CK vs. L1, which included 4191 upregulated genes and 612 downregulated genes. Out of 3355 genes differentially expressed in CK vs. L3, 972 were upregulated and 2383 were downregulated. Likewise, out of 9388 genes differentially expressed in CK vs. L3, 7561 were upregulated and 1827 were downregulated (Figure 4A). The Venn diagram of DEGs in CK vs. L1, CK vs. L2, and CK vs. L3 had a total of 493 overlapping genes in all comparisons that were controlled by different levels of shade (Figure 4B). Furthermore, 2744, 2125, and 6904 specific genes were revealed by the Venn diagram in CK vs. L1, CK vs. L2, and CK vs. L3, respectively.

#### 2.2.4. KEGG Enrichment of Differentially Expressed Genes

**Figure 5:**
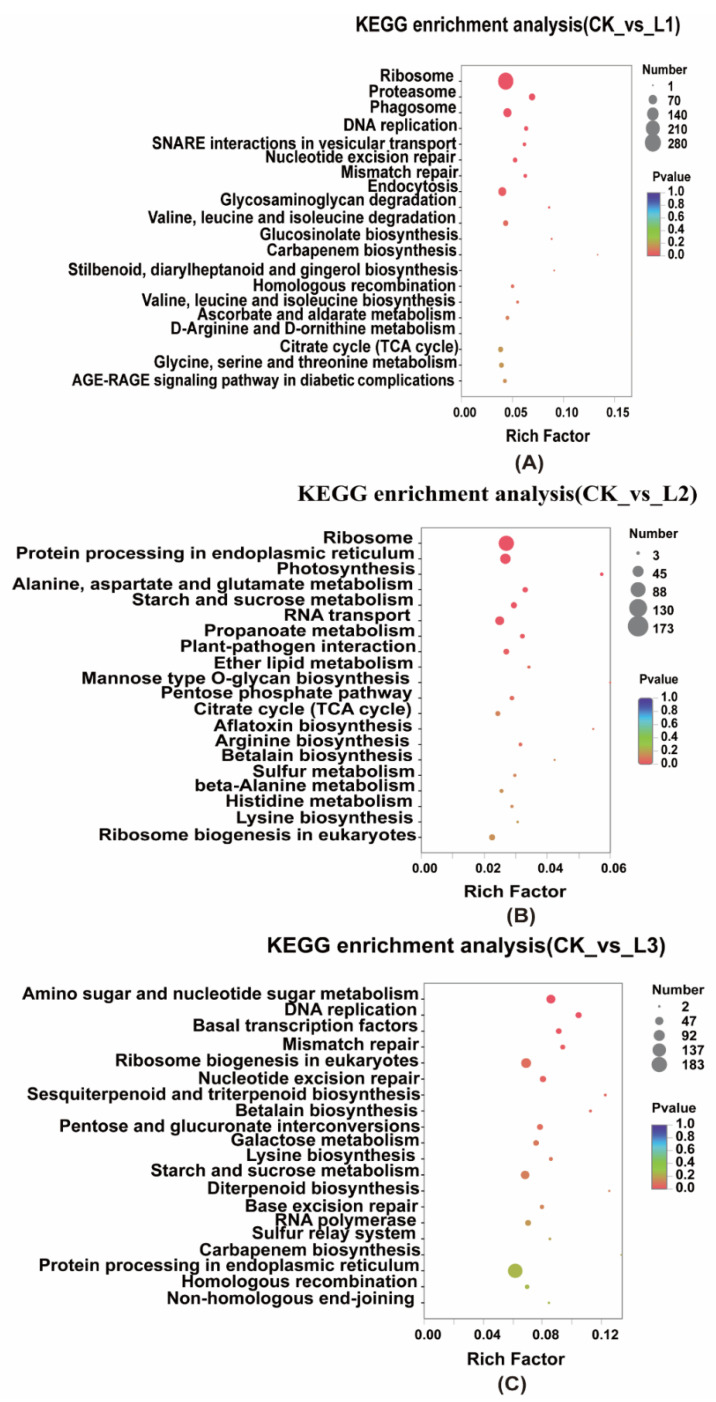
KEGG enrichment analysis of DEGs of root tissues *A. dahurica* under different light intensities. (**A**) CK vs. L1; (**B**) CK vs. L2; (**C**) CK vs. L3.

We performed KEGG pathway enrichment analysis of the DEGs (Figure 5) in roots under different shade conditions. In both CK vs. L1 and CK vs. L2, the DEGs were enriched for “Ribosome”. In addition, “Proteasome”, “DNA replication”, “Endocytosis”, “DNA mismatch repair”, “Nucleotide excision repair”, “SNARE interactions in vesicular transport”, and “Glycosaminoglycan degradation” were significantly enriched in CK vs. L1 (Figure 5A). In CK vs. L2, significant enrichment in “Ribosome”, “Protein processing in the endoplasmic reticulum”, “RNA transport”, “plant-pathogen interaction”, “Propanoate metabolism”, “Starch and sucrose metabolism”, “Alanine, aspartate, and glutamate metabolism”, and “photosynthesis” (Figure 5B) was found. In CK vs. L3, significant enrichment in “amino sugar and nucleotide sugar metabolism” and “DNA replication” was observed (Figure 5C).

#### 2.2.5. Coumarin Biosynthesis Pathway

**Figure 6:**
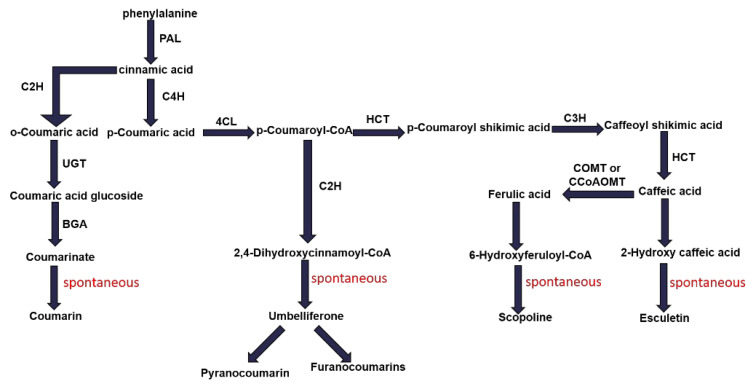
Expression patterns of genes involved in coumarin biosynthesis.

**Figure 7:**
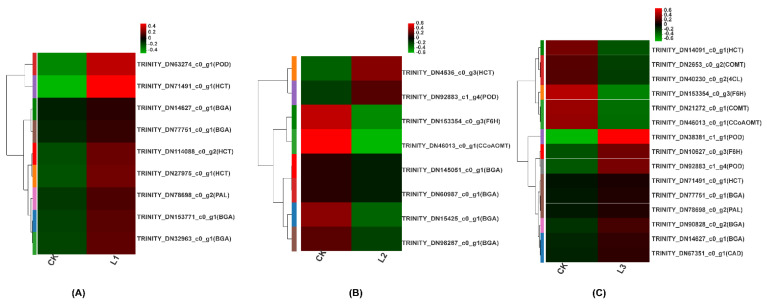
Heat map of differential expression of the genes in the coumarin biosynthesis pathway under different light intensities. (**A**) CK vs. L1; (**B**) CK vs. L2; (**C**) CK vs. L3. Red and green colors correspond to high and low expression levels, respectively.

Coumarins are secondary metabolites that have important pharmacological effects and are synthesized by the phenylpropanoid biosynthetic pathway (Figure 6). We further investigated the expression of the genes in the phenylpropanoid pathway involved in coumarin biosynthesis under different shade conditions. We identified the enrichment of 9 genes (4 BGA, 3 HCT, 1 POD, 1 PAL) in CK vs. L1, 8 genes (1 POD, 1 HCT, 4 BGA, 1 CCoAOMT, 1 F6H) in CK vs. L2, and 15 genes (3 BGA, 2 POD, 2 HCT, 2 F6H, 2 COMT, 1 4CL, 1 PAL, 1 CAD, 1 CCoAOMT) in CK vs. L3 (Figure 7). In CK vs. L1, all nine differentially expressed genes were upregulated (Figure 7A). In CK vs. L2, two genes were upregulated and six were downregulated, whereas in CK vs. L3, nine genes were upregulated and six were downregulated (Figure 7B). Overall, 4CL (TRINITY_ DN40230_c0_g2) and COMT (TRINITY_DN21272_c0_g1) genes were most significantly downregulated (Figure 7C).

#### 2.2.6. qRT-PCR Validation

**Figure 8:**
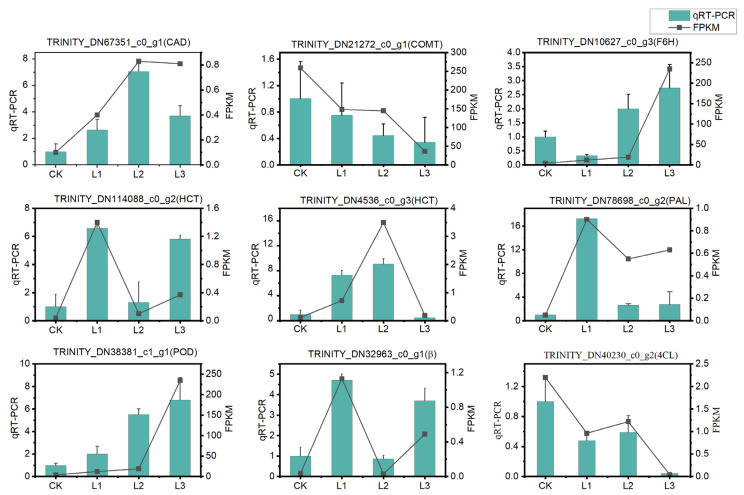
The expression of nine randomly selected DEGs was determined by RNA-seq and qRT-PCR under different light intensities. The y-axis on the left indicates the qRT-PCR expression levels and the y-axis on the right indicates the RNA-seq data with Log2 (FPKM). The x-axis indicates the light treatments.

To validate the RNA-seq data, qRT-PCR was used to detect and quantify POD (TRINTY_DN38381_c1_g1), BGA (TRINITY_DN32963_c0_g1), CAD (TRINITY_DN67351_c0_g1), COMT (TRINITY_DN21272_c0_g1), F6H (TRINITY_DN10627_c0_g3), HCT (TRINITY_ DN114088_c0_g2, TRINITY_DN4536_c0_g3), PAL (TRINITY_DN78698_c0_g2), and 4CL (TRINITY_ DN40230_c0_g2) unigenes. Out of 10 genes, 9 genes showed the same expression trend as RNA-seq (Figure 8). In conclusion, the qRT-PCR results indicate that the RNA-Seq data are reliable.

## 3. Materials and Methods

### 3.1. Plant Materials and Experimental Design

The seeds of *A. dahurica* were purchased from the Kangmei Chinese herb market in Bozhou City, Anhui Province, and were sown at the Zheshan Campus of Anhui Normal University, Wuhu City, Anhui Province (31°33′ N, 118°36′ E). For the pot experiments, topsoil (0–20 cm) was collected from the campus of Anhui Normal University. The physicochemical properties of the soil are listed in Supplementary Table S7.

After 3 months of sowing, three homogeneous seedlings were transplanted into plastic pots (height, 17 cm and inner diameter, 15 cm) containing 2 kg of air-dried soil. We established an experimental area of potted plants with different light intensities by covering various subareas of the experimental area with black shade nets of different thicknesses (encrypted with four needles). A digital lux meter (TES-1336A, TES Electrical Electronic Corp., Taiwan) was used to photosynthetically measure active radiation at 11:00 a.m. and 3:00 p.m. on the day of the measurement. For each light treatment, 15 pots were prepared, and all experiments were performed three times. The following four light treatments were set up in the experiment: natural light (CK, 18,417 ± 228.11 Lux/Fc); shade rate 50% (L1, 8607 ± 217.79 Lux/Fc); shade rate 70% (L2, 5227 ± 70.24 Lux/Fc); and shade rate 90% (L3, 2183 ± 40.42 Lux/Fc). Light treatment was followed by the collection of leaves and roots, which were immediately frozen in liquid nitrogen and stored at −80 °C for the subsequent determination of physiological indexes, transcriptome sequencing, and qRT-PCR experiments.

### 3.2. Measurement of Physiological Indicators

The physiological indicators, including chlorophyll content, malondialdehyde concentration (MDA), superoxide dismutase (SOD) activity, peroxidase (POD) activity, catalase (CAT) activity, total free proline (Pro) content, soluble protein (SP) content, and soluble sugar (SS) content were determined according to the method of Li [25].

### 3.3. cDNA Library Construction and Functional Annotation

Root tissue RNA was extracted using a TRIzol^®^ Reagent (Invitrogen, Carlsbad, CA, USA) kit. The concentration, purity, and integrity of RNA were analyzed by NanoDrop2000 (Thermo Scientific, Waltham, MA, USA) and Agilent 2100 (Agilent Technologies, Santa Clara, CA, USA) bioanalyzer methods. The mRNA was fragmented into approximately 200 bp fragments, and the first-strand and second-strand cDNA were synthesized using magnetic beads with Oligo (dT). The fragments were separated by agarose gel electrophoresis and used for PCR amplification to generate sequencing templates. Samples were sequenced on an Illumina Novaseq 6000 platform (Majorbio, Shanghai, China) with a PE 150 read length.

The raw data were evaluated and filtered by spurious sequence removal and the removal of redundancy by Sickle and SeqPrep [26] to obtain clean reads. De novo splicing and assembly were performed using Trinity [27]. TransRate [28] and CD-HIT [29] were used to perform the quality analysis of the de novo transcriptome assembly and reduce sequence redundancy to finally obtain unigenes. The assembled unigenes were functionally annotated with the following six major public databases: Gene Ontology (GO); Kyoto Encyclopedia of Genes and Genomes (KEGG); evolutionary genealogy of genes: non-supervised orthologous groups (eggNOG); non-redundant protein sequence (NR); RefSeq non-redundant proteins (Swiss-Prot); and Protein families (Pfam).

### 3.4. Expression Analysis of Differentially Expressed Genes

Gene expression levels were quantified using RSEM [30], and the differential expression ploidy of the genes between the samples was calculated according to the FPKM [31] method. The screening of differentially expressed genes between the groups was performed using DESeq2 [32] with |log2Fold-change| ≥ 2 and *p*-value < 0.05 as the screening criteria. Gene sets of differentially expressed genes between different treatments were constructed and the differentially expressed genes were subjected to GO and KEGG functional classification and enrichment analysis. Coumarin biosynthetic genes were identified by BLAST [33] based on the coumarin biosynthetic pathway, combined with the KEGG annotation results and known gene information in the database.

### 3.5. Real-Time Quantitative PCR Validation

Total RNA was extracted using the SteadyPure Plant RNA Extraction Kit (AG21019 Accurate Biotechnology, Changsha, Hunan, China) as per the manufacturer’s instructions. cDNA was synthesized by reverse transcription using the Evo M-MLV RT Kit (AG11728, Accurate Biotechnology, Changsha, Hunan, China). Real-time quantitative PCR (qRT-PCR) was performed using SYBR^®^ Green Premix pro Taq HS qPCR Kit (AG11701 Accurate Biotechnology, Changsha, Hunan, China) in a 20 μL reaction volume that contained 2 μL cDNA, 0.4 μL 2 μM forward primer, 0.4 μL 2 μM reverse primer, 0.4 μL ROX Reference Dye (50X), 10 μL 2 × SYBR Green Premix Premix pro Taq, and 6.8 μL RNase-free water. Ten candidate genes in the coumarin biosynthetic pathway were selected for qRT-PCR validation, and the actin gene [13] was used as an internal reference control gene. The specific primers for the qRT-PCR of the genes are shown in (Supplementary Table S8), and the qPCR amplification conditions were as follows: 95 °C for 30 s; 40 cycles at 95 °C for 5 s; and 60 °C for 30 s. Relative expression was calculated using the 2^−ΔΔCt^ method [34], and the results were obtained for each gene using three biological replicates and three technical replicates of each sample.

### 3.6. Statistical Analysis

Origin 2021 software was used to plot the data. SPSS 19.0 software was used for one-way ANOVA and Duncan’s method of multiple comparisons (*p* < 0.05). The data are expressed as mean ± standard deviation (SD).

## 4. Discussion

### 4.1. Effect of Different Light Intensities on Physiological Characteristics of Leaves

It is essential for plants to produce photosynthetic pigments, and the contents of pigments in the leaves indicate a plant’s level of photosynthetic capacity [35]. Our study showed that the contents of Chl a, Chl b, Chl a + b, and Chl a/b differed significantly between treatments in *A. dahurica* seedlings (Figure 1). With increasing shade intensity, the contents of Chl a, Chl b, and Chl a + b increased, indicating that *A. dahurica* seedlings increased their pigment content to capture more light energy and improve photosynthetic efficiency [36]. Bertamini et al. [37] found that plants in deep shade had a reduced chlorophyll a/b ratio and enhanced the use of blue-violet light to adapt to shady conditions. As a result of our study, the Chl a/b ratio decreased with increasing shade intensity compared to other shade treatments, implying that the decrease in the Chl a/b ratio might be an adaptation mechanism for *A. dahurica* seedlings to lower light conditions [38].

As a consequence of biotic and abiotic stresses, reactive oxygen species (ROS) are produced in high contents [39]. Our results showed that SOD and CAT activity both increased with decreasing light intensity (Figure 2), indicating that SOD, as the first line of defense in the reactive-oxygen-species-scavenging enzymatic system, helps plants cope with low-light stress by generating a stress response [40]. When CAT activity increases, H_2_O_2_ is broken down into H_2_O and O_2_, protecting the cell membrane structure by restoring the balance between free radical production and elimination [41]. In contrast with our findings, Zhu et al. [42] and Deng et al. [43] observed a decrease in ROS production when ROS was not cleared. When lipid peroxidation is induced by adverse stress, MDA is a key indicator of cellular damage [44]. With the increase in shading rate, MDA content decreased and then increased, and was lowest at 50% shade (L1), indicating too strong or too weak light stressed the *A. dahurica* seedlings. This finding is consistent with the results reported by Gao et al. [45].

SS, SP, and Pro are important substances involved in plant metabolism and are also important osmoregulatory substances. As plants grow in unsuitable light environments, a decrease in intracellular osmotic pressure occurs and plants respond by increasing the concentration of intracellular solutes [46]. Based on our results, SS levels increased in the L1 treatment group, and then decreased with the decrease in light intensity in the L2 and L3 groups. They were significantly higher in the L1 group than in the other treatment groups (Figure 3). As a result of shade treatment, sugar synthesis declined, respiration increased, and photosynthetic products were consumed [47]. The content of SP and Pro decreased gradually, which may be due to the lower photosynthetic rate and the inhibition of soluble protein accumulation in the plant leaves under the weaker light intensity levels, leading to decreased protein content in the leaves [48].

### 4.2. Effect of Different Light Intensities on the Transcriptome of Roots

The biomass of the belowground part of a plant and the accumulation of secondary metabolites are closely related to the photosynthetic efficiency of the aboveground part. The biosynthesis of secondary metabolites is influenced not only by the amount of original carbon skeletal material, but also by the activity of enzymes in the biosynthetic pathway [49]. In this study, we examined the expression of a subset of coumarin biosynthesis pathway genes in the roots of *A. dahurica* after shading the aboveground parts. we used the Illumina Novaseq 6000 sequencing platform for RNA-Seq analysis and obtained 294,362 unigenes with an average length of 823.54 bp, N50 length of 1331 bp, and GC content of 42.79% (Supplementary Figure S1, Supplementary Table S1 and Supplementary Table S2). This indicated a good coverage of the root transcriptome, and a total of 127,670 unigenes genes were annotated in the six databases (Supplementary Table S3). A search in the NR database revealed that the most closely related species based on gene sequences were *Daucus carota* (Supplementary Figure S4).

As shown in Figure 4, a total of 4803 genes were differentially expressed in CK vs. L1 (4191 upregulated and 612 downregulated), 3355 in CK vs. L2 (972 upregulated and 2383 downregulated), and 9388 in CK vs. L3 (7561 upregulated and 1827 downregulated). The results showed that the number of differentially expressed genes was the highest in CK vs. L3, indicating that L3 was most affected by low-light stress. The number of genes upregulated was greater than the number of genes that downregulated light stress regulation in CK vs. L1 and CK vs. L3. Only in CK vs. L2 was the number of upregulated genes of smaller than the downregulated genes. This indicates that the roots of *A. dahurica* mainly adapted to light stress through the positive regulation of genes [50].

Based on the DEG KEGG enrichment results, CK vs. L1 and CK vs. L2 were significantly enriched in “Ribosomes,” suggesting that shade at the level of L1 and L2 treatments may contribute to protein synthesis and that ribosomes have positive effects on plant reproduction, suitable for growth [51] (Figure 5A,B). In contrast, the KEGG enrichment of CK vs. L3 was significantly higher in “Amino sugar and nucleotide sugar metabolism” (Figure 5C), indicating that shade influenced the regulation of amino and nucleotide sugar metabolism in the plants in the stressful environment of the L3 group. Changes in the “Amino sugar and nucleotide sugar metabolism” may affect the expression and regulation of the activity of the β-glucosidase enzyme in the coumarin biosynthesis pathway [52].

### 4.3. Effect of Different Light Intensities on Coumarin Synthesis Pathway of Roots

Many studies have demonstrated that coumarin biosynthesis takes place through the phenylpropanoid metabolic pathway, a major source of several defensive secondary metabolites in plants that plays a crucial role in protecting plants from microbial infection, herbivore predation, and environmental stress [53]. The phenylpropanoid metabolic pathway is derived from the carbon skeleton of phenylalanine [22]. Phenylalanine ammonia-lyase (PAL), Cinnamate-4-hydroxylase (C4H), and 4-coumarate-CoA ligase (4CL) are the main enzymes in the phenylpropanoid metabolic pathway [54,55]. PAL is the first enzyme in the pathway that catalyzes the production of trans-cinnamic acid from phenylalanine, which is subsequently converted to many phenylpropane compounds (Figure 6), such as lignans, anthocyanins, flavonoids, and coumarins, through a series of catalytic reactions in the presence of C4H and 4CL enzymes [56,57]. We identified 10 genes in this study, i.e., PAL, C4H, 4CL, HCT, F6H, CAD, POD, CCoAOMT, COMT, and BGA, which are part of the phenylpropanoid metabolic pathway (Figure 7). The PAL (TRINITY_DN78698_c0_g2) gene was significantly upregulated in the L1 treatment group compared with CK (Figure 7A), indicating that, at 50% shade, higher transcript levels of this gene may lead to higher levels of coumarins [58]. 4CL is a key component of the plant phenylpropanoid pathway, including the coumarin biosynthesis pathway, and is required for the synthesis of major coumarins in plants [59,60]. We found that, compared with the CK group, the 4CL gene (TRINITY_DN40230_c0_g2) was significantly downregulated in the L3 group, suggesting that a high degree of shading, as in the L3 group, may decrease coumarin biosynthesis by regulating the 4CL gene (Figure 7C).

Previous research by Luo et al. [52] and Zhao et al. [61] demonstrated the catalytic mechanism of COMT in coumarin biosynthesis and proved its importance as a critical enzyme in the coumarin biosynthesis pathway. Kai et al. [62] reported that the content of coumarin in *Arabidopsis* decreased when COMT was mutated. In our study, COMT genes (TRINITY_DN21272_c0_g1) were found to be significantly downregulated in the L3 group (Figure 7C), implying coumarin biosynthesis may be decreased at the 90% shade level of the L3 group [13]. The downregulation of COMT (TRINITY_DN21272_c0_g1) was verified by qRT-PCR and was consistent with the results of RNA-seq (Figure 8).

## 5. Conclusions

In this study, we analyzed the leaf and root tissues of seedlings under full light (CK), 50% shading (L1), 70% shading (L2), and 90% shading (L3). The changes in the leaf physiological indexes showed that shades of 50% (L1) and 70% (L2) improved chlorophyll synthesis and increased photosynthesis. Antioxidant enzyme activity produced a stress response with the decrease in the light intensity, and MDA increased due to membrane lipid peroxidation in full light (CK) and shade of 90% (L3), indicating that lipid peroxidation is affected by both high- and low-intensity light. Osmoregulatory substances were also affected by light intensity. SS accumulated most in the L1 treatment group, and both SP and Pro were inhibited by shading. Thus, 50% shade is favorable for the growth and development of seedlings. Furthermore, transcriptome analysis revealed that the root tissues had a significant number of differentially expressed genes under various shade treatments. We discovered that genes involved in coumarin synthesis were influenced by light, as demonstrated by the upregulation and downregulation of the genes in the phenylpropanoid metabolic pathway. The PAL (TRINITY_DN78698_c0_g2) gene, which is upstream of the coumarin biosynthetic pathway, was significantly upregulated at 50% shade (L1), suggesting that coumarin synthesis is promoted in this environment. At 90% shade (L3), the upstream gene 4CL (TRINITY_DN40230_c0_g2) and the downstream gene COMT (TRINITY_DN21272_c0_g1) were significantly downregulated, indicating that coumarin synthesis may be significantly inhibited under low-light conditions. The results of this study can be used to improve the yield of the secondary metabolites and quality of *A. dahurica* and other medicinal plants.

## Figures and Tables

**Table 1:** Glossary of abbreviations for professional terms.

Abbreviations	Terminology	Abbreviations	Terminology
*A. dahurica*	*Angelica dahurica* (Fisch.ex Hoffm.) Benth. et Hook. f.	Chl b	Chlorophyll b
PAL	Phenylalanine ammonia-lyase	Chl a + b	Chlorophyll a + b
C4H	Cinnamic acid-4-hydroxylase	Chl a/b	Chlorophyll a/b
4CL	4-coumaric acid-CoA ligase	MDA	Malondialdehyde concentration
HCT	Hydroxycinnamic acid acyltransferase	SOD	Superoxide dismutase
C3H	P-coumaric acid 3-hydroxylase	POD	peroxidase
COMT	Caffeic acid O-methyltransferase	CAT	Catalase
CCoAOMT	Caffeoyl CoA O-methyltransferase	Pro	Free proline
BGA	β-glucosidase	SP	Soluble protein
F6H	Feruloyl-CoA 6-hydroxylase	SS	Soluble sugar
CAD	Coniferyl-aldehyde dehydrogenase	GO	Gene Ontology
C2H	Cinnamic acid 2-hydroxylase	KEGG	Kyoto Encyclopedia of Genes and Genomes
qRT-PCR	Real-time quantitative Polymerase Chain Reaction	eggNOG	Non-supervised orthologous groups
RNA-seq	RNA-sequencing	NR	Non-redundant protein sequence
RSEM	RNA-Seq by Expectation-Maximization	Swiss-Prot	RefSeq non-redundant proteins
Chl a	Chlorophyll a	Pfam	Protein families

## Data Availability

The sequence data have been uploaded to the SRA database of the National Center for Biotechnology Information under the accession number PRJNA883999.

## Supplementary Materials

Supplementary Materials can be downloaded at: https://www.mdpi.com/article/10.3390/ijms232415912/s1.
