# Supplementary material for: Effects of Light Intensity on Physiological Characteristics and Expression of Genes in Coumarin Biosynthetic Pathway of Angelica dahurica
Source: Int J Mol Sci. 2022 Dec 14;23(24):15912. doi: 10.3390/ijms232415912 (PMC9781474; doi:10.3390/ijms232415912)
Supplement: Supplementary file 1 [file ijms-23-15912-s001.zip › Figure S2. GO annotation.pdf]

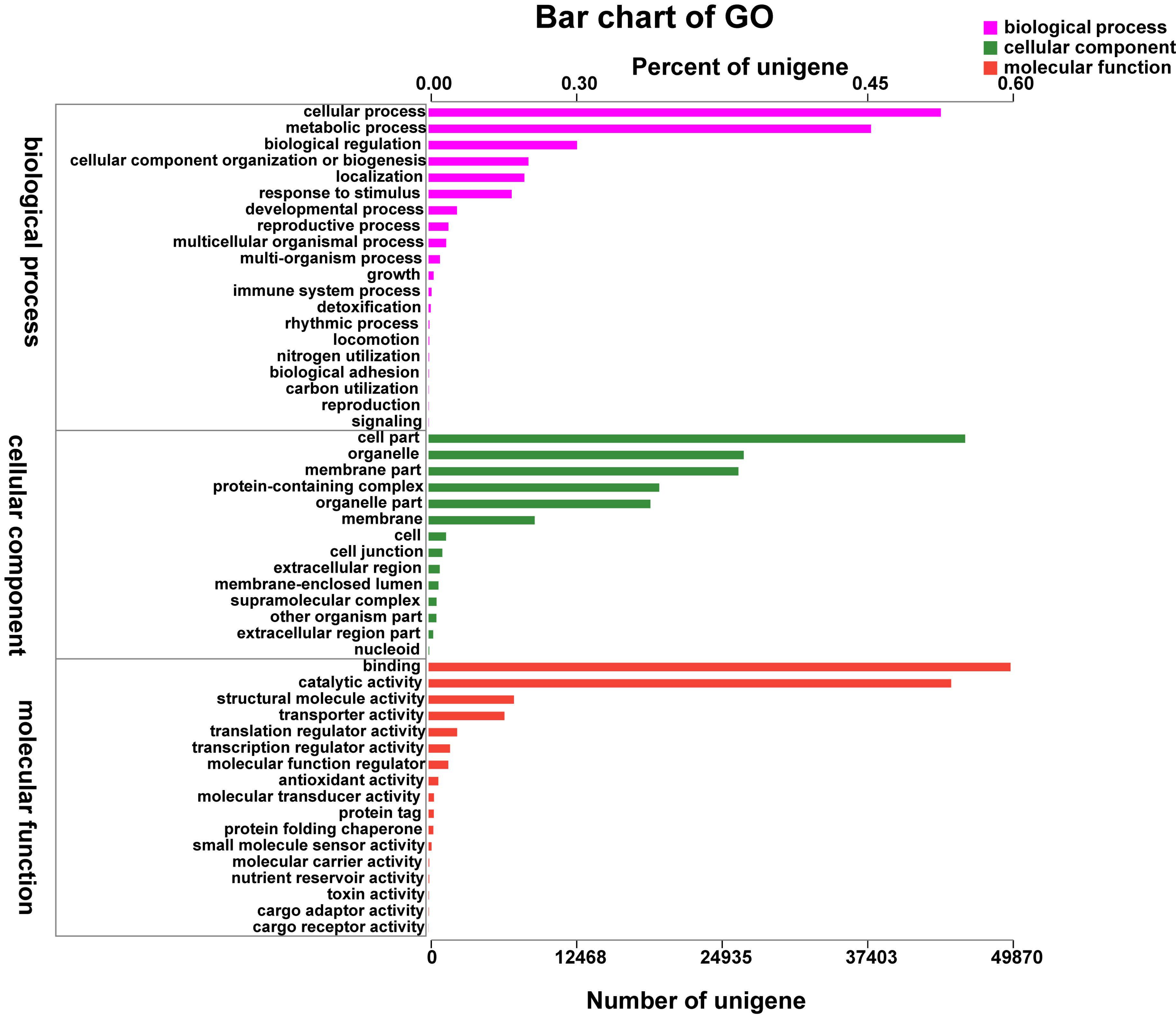
Bar chart of GO
biological process
cellular component
molecular function
Percent of unigene
0.00
0.30
0.45
0.60
biological process
cellular process
metabolic process
biological regulation
cellular component organization or biogenesis
localization
response to stimulus
developmental process
reproductive process
multicellular organismal process
multi-organism process
growth
immune system process
detoxification
rhythmic process
locomotion
nitrogen utilization
biological adhesion
carbon utilization
reproduction
signaling
cellular component
cell part
organelle
membrane part
protein-containing complex
organelle part
membrane
cell
cell junction
extracellular region
membrane-enclosed lumen
supramolecular complex
other organism part
extracellular region part
nucleoid
molecular function
binding
catalytic activity
structural molecule activity
transporter activity
translation regulator activity
transcription regulator activity
molecular function regulator
antioxidant activity
molecular transducer activity
protein tag
protein folding chaperone
small molecule sensor activity
molecular carrier activity
nutrient reservoir activity
toxin activity
cargo adaptor activity
cargo receptor activity
0
12468
24935
37403
49870
Number of unigene
