# Supplementary material for: Effects of Light Intensity on Physiological Characteristics and Expression of Genes in Coumarin Biosynthetic Pathway of Angelica dahurica
Source: Int J Mol Sci. 2022 Dec 14;23(24):15912. doi: 10.3390/ijms232415912 (PMC9781474; doi:10.3390/ijms232415912)
Supplement: Supplementary file 1 [file ijms-23-15912-s001.zip › Supplemetary Tables S1, S2, S3, S7, S8.pdf]

International Journal of
*Molecular Sciences*

MDPI

## Supplementary Materials

**Table S1.** Sequencing data of *A. dahurica* roots in different shading treatment.

| Sample | Clean reads | Clean base | GC Content | Q30(%) |
|---|---|---|---|---|
| CK-1 | 43958162 | 6349617042 | 43.34 | 93.72 |
| CK-2 | 43881308 | 6334760866 | 44.03 | 93.87 |
| CK-3 | 59613146 | 8579749179 | 42.31 | 94.2 |
| L1-1 | 42026396 | 8579749179 | 43.44 | 93.72 |
| L1-2 | 43277064 | 6251278155 | 43.42 | 93.9 |
| L1-3 | 42981158 | 6242766797 | 43.4 | 93.78 |
| L2-1 | 48032450 | 7049899910 | 43.4 | 93.81 |
| L2-2 | 51661628 | 7598466542 | 43.67 | 93.85 |
| L2-3 | 51300598 | 7443134197 | 44.4 | 93.88 |
| L3-1 | 42632148 | 6216459055 | 44.08 | 93.57 |
| L3-2 | 49058844 | 7138566211 | 43.63 | 93.99 |
| L3-3 | 48815108 | 7139405562 | 43.74 | 93.82 |

**Table S2.** Assessment of the results of root *de novo* assembly of *A. dahurica* in different shading treatment.

| Type | Unigene |
|---|---|
| Total number | 294362 |
| Total base | 242418069 |
| Largest length (bp) | 12050 |
| Smallest length (bp) | 201 |
| Average length (bp) | 823.54 |
| N50 length (bp) | 1331 |
| E90N50 length (bp) | 1955 |
| Fragment mapped | 87.912 |
| GC percent (%) | 42.79 |

**Table S3.** Summary statistics of the annotations for *A. dahurica* root unigenes from six public databases.

| Database | Number of unigenes | Percent（%） |
|---|---|---|
| GO | 83018 | 41.02% |
| KEGG | 70589 | 34.88% |
| eggNOG | 100463 | 49.64% |
| NR | 101839 | 50.32% |
| Swiss-Prot | 89172 | 44.06% |
| Pfam | 95434 | 47.16% |
| Total annotated | 127670 | 63.09% |

**Table S7.** Basic physical and chemical properties of the tested soil.

| Soil type | SOM（$g·kg^{-1}$） | TN（$mg·kg^{-1}$） | TP（$g·kg^{-1}$） | TP（$g·kg^{1}$） |
|---|---|---|---|---|
| Content | 12.25 | 1.15 | 0.13 | 10.28 |

**Table S8.** Gene and primers used for the qRT-PCR validation.

| Genes | Former primer (5′-3′) | Back primer (5′-3′) |
|---|---|---|
| actin | GATTCCGTTGCCCTGAGGTTCTG | ACCACCACTGAGCAC-TATGTTTCC |
| TRINITY_DN67351_c0_g1 | TTTTATGTCCGCATTATCAGG | CGGGTTTCTG-TACAAAATCAGAC |
| TRINITY_DN21272_c0_g1 | TATTAAAGGCATCAACTTCGAC | CTTCAAGAATATGGCGTCTC |
| TRINITY_DN10627_c0_g3 | GAAATTATTGGTCCGCTACCTG | ATAATCCGAATACAAAACTT-GCT |
| TRINITY_DN114088_c0_g2 | AAGTAAACAGTGAACACGCAAA | TCTGCACCAACTAGTACACC |
| TRINITY_DN4536_c0_g3 | TTACATGATCGCCTGACGCCAA | CTTAGCCTTCCAGCAAATGGGT |
| TRINITY_DN78698_c0_g2 | GATTCCGTTGCCCTGAGGTTCTG | ACCACCACTGAGCAC-TATGTTTCC |
| TRINITY_DN38381_c1_g1 | TAATCCGACAGCATTCTTGGAC | AAATTTGAAACTATCGGAGCAC |
| TRINITY_DN32963_c0_g1 | CGCCTTCCTAGCTTTTCGG | AGCTGTAATGGTTAAGCCCAA |
| TRINITY_DN40230_c0_g2 | ATTGCACCACCTGTGTTCGATG | ATCATTTGCGTCCAAGTAAGCC |
