# Supplementary figures and images for: Effects of Light Intensity on Physiological Characteristics and Expression of Genes in Coumarin Biosynthetic Pathway of Angelica dahurica
Source: Int J Mol Sci. 2022 Dec 14;23(24):15912. doi: 10.3390/ijms232415912 (PMC9781474; doi:10.3390/ijms232415912)

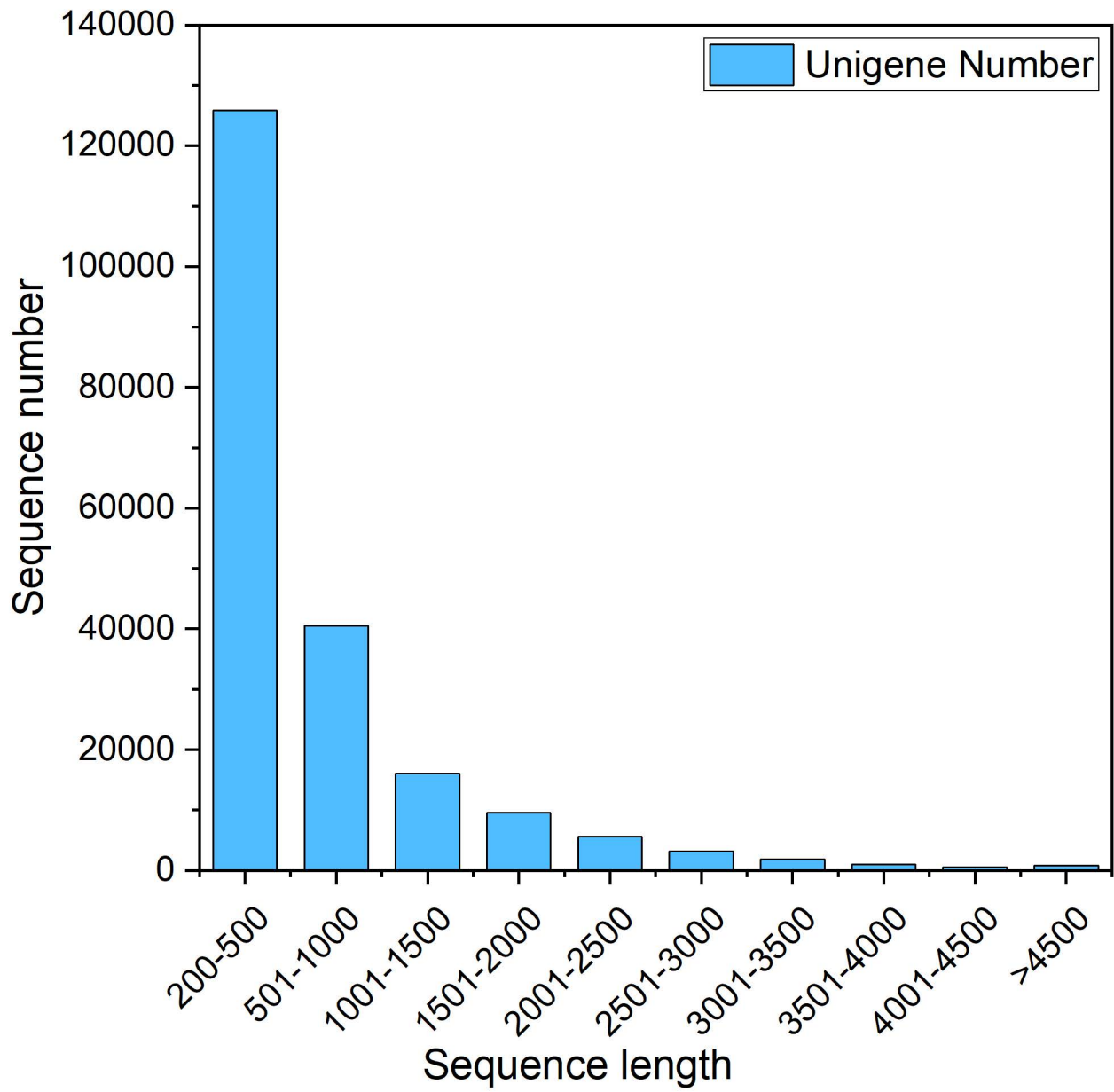

Supplement: Supplementary file 1 [file ijms-23-15912-s001.zip › Figure S1. Length distribution of unigenes.pdf]

# Gene functional classification (KEGG)

Number of unigenes

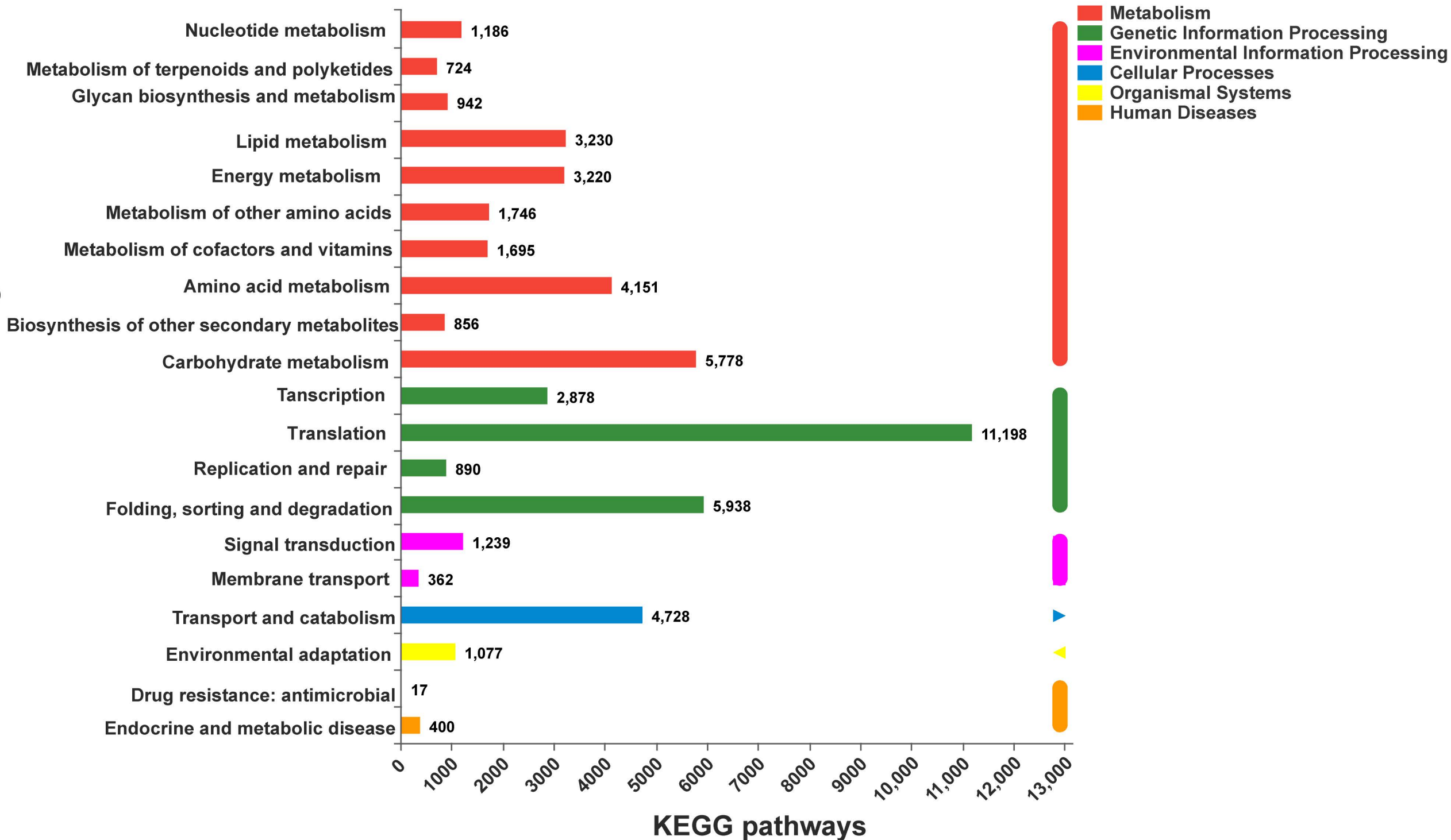

Supplement: Supplementary file 1 [file ijms-23-15912-s001.zip › Figure S3 KEGG annotation.pdf]

# Species Distribution

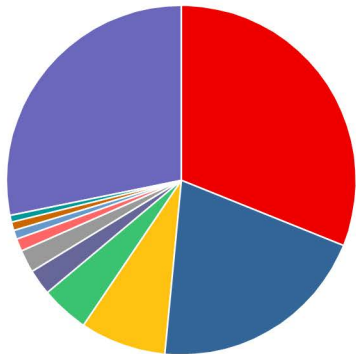

# Specices

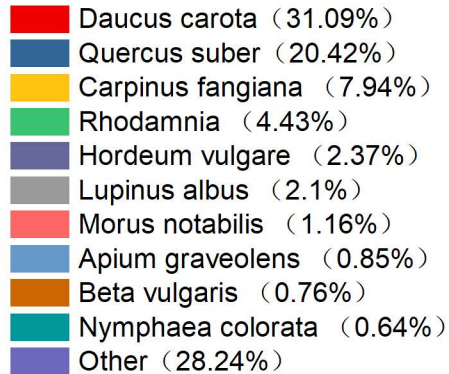

Supplement: Supplementary file 1 [file ijms-23-15912-s001.zip › Figure S4 NR annotation.pdf]
